# Prevalence of overweight/obesity and its associated factors among a sample of Moroccan type 2 diabetes patients

**DOI:** 10.4314/ahs.v21i1.5

**Published:** 2021-03

**Authors:** Ahmed Chetoui, Kamal Kaoutar, Keltoum Boutahar, Abdeslam El Kardoudi, Soufiane Elmoussaoui, Fatiha Chigr, Mohamed Najimi

**Affiliations:** 1 Biological Engineering Laboratory, Faculty of Sciences and Techniques, Sultan Moulay Slimane University, Beni Mellal, Morocco; 2 Mohamed VI Hospital University, Marrakesh, Morocco

**Keywords:** Obesity, overweight, abdominal obesity, type 2 diabetes, Morocco

## Abstract

**Background:**

Obesity constitutes a major risk factor for the development of diabetes, and has been linked with poor glycaemic control among type 2 diabetic patients.

**Aims:**

This study examines the prevalence of overweight/obesity and associated factors in type 2 diabetic patients in the Beni-Mellal Khenifra region in Morocco.

**Methods:**

A questionnaire-based cross-sectional study was conducted in 2017 among 975 diabetes patients attending primary health centres. Demographic and clinical data were collected through face-to-face interviews. Anthropometric measurements, including body weight, height and waist circumference, were taken using standardized techniques and calibrated equipment.

**Results:**

The prevalence of overweight was 40.4%, the general obesity was 28.8% and the abdominal obesity was 73.7%. Using multivariate analysis, we noted that the general obesity was associated with female sex (AOR= 3,004, 95% CI: 1.761–5.104, P<0.001), increased age (AOR=2.192, 95% CI: 1.116–4.307, P<0.023) and good glycaemic control (AOR=1.594, 95% CI: 1.056–2.407, P=0.027), whereas abdominal obesity was associated wih female sex (AOR=2.654, 95% CI: 1.507–4.671, P<0.001) and insulin treatment (AOR=2.927, 95% CI: 1.031–8.757, P=0.048).

**Conclusion:**

Overweight, general obesity and abdominal obesity were high among participants, especially among women. Taken together, these findings urge the implementation of a roadmap for this diabetic subpopulation to have a new lifestyle.

## Introduction

Diabetes is a major public health problem due to its negative effects on health and well-being and the costs engendered by its complications. This could be exacerbated by the fact that the prevalence of this disease is elevated and is highly dynamic with a serious and socioeconomic impact^1^. As a result, diabetes is currently one of the most worrying diseases in both industrialised and developing countries, as the latter are in nutrition transition.

According to the International Diabetes Federation^2^, the number of adult diabetic patients recorded in 2015 was 415 million, representing 8.8% of the world's population, and type 2 diabetes mellitus (T2DM) is the most common form of diabetes, representing more than 90% of all declared cases^3^. In Morocco, a country in the full phase of demographic, nutritional and epidemiological transition^4^,^5^, diabetes is emerging as a preoccupying public health issue that represents a challenge facing health practitioners daily. Based on a survey carried out by health authorities in 2000, the prevalence of diabetes in Morocco was about 6.6% in a population aged at least 20 years 6, whereas it reached 10.6% in 2018 according to the latest national survey on the common risk factors for non-communicable diseases^7^. These statistics reflect the evolution of the disease in Morocco and that the situation requires rigorous management of diabetes along with its associated factors such as overweight and obesity.

It is well known that for diabetic and non-diabetic persons, being overweight or obese is a major risk factor. That is why professional organisations and health professionals recommend weight loss as a primary strategy for glycaemic control. For example, the American Diabetes Association (ADA) recommends weight loss for all overweight or obese individuals who have or are at risk for diabetes^8^.

There is a very close relation between weight and T2DM. Indeed, many previous published data have reported that most of patients with T2DM are overweight or obese and that obese people present the highest risk of developing T2DM^9^. The simultaneous occurrence of the complicated conditions of diabetes and obesity within a single individual is called ‘diabesity’^10^. Furthermore, overweight and obesity in diabetics are associated with poorer control of blood glucose levels and blood pressure^11^, which represents high cardiovascular risk^12^. Conversely, intentional weight loss is associated with reduced mortality in people with ‘diabesity’ 13.

Determinants of weight gain leading to overweight and obesity are clearly multifactorial and involve genetic, socioeconomic and environmental components14. Additionally, assessment of the nutritional status of patients with T2DM is essential to detect malnutrition that could increase morbidity and mortality and prolong the length of hospital stay. However, data regarding nutritional status and its associated factors in Moroccan type 2 diabetic patients remain scarce and limited. To our knowledge, there are no published studies on this subject in the Beni-Mellal Khenifra (BM-KH) region and in other regions of Morocco, except the study carried out by Ramdani et al. in 2012 on diabetes and obesity in eastern Morocco^15^. A better understanding of the factors associated with diabesity in the Moroccan context is strongly needed to help diabetics and health professionals better manage diabetes. Thus, the aim of this work is to determine the prevalence of overweight and obesity and to identify its determinant factors in a sample of type 2 diabetic patients in the BM-KH region.

## Methods

### Study participants and data collection

We conducted a cross-sectional survey in 2017 among 975 T2DM patients attending primary health centres in the BM-KH region of Morocco. At the time of the survey and according to the Regional Observatory of Health in the BM-KH region, the primary health centres provide health services for 153 000 T2DM patients registered in five provinces who receive regular medical follow-up and get their medications dispensed at the centres free of charge.

For patient selection, a multilevel random-sampling method was used to recruit participants.

The sample size was calculated based on the following parameters: prevalence of overweight and obesity (50%) among T2DM patients, 4% margin of error (e=0.04) and 95% confidence level (z=3.20); thus, the minimum study sample size was 932, which was rounded up to 1000 persons for more accuracy and in order to account for possible exclusions and the need to carry out subgroup analysis.

The sample surveyed in the five provinces of the BMKH region was proportional to the total T2DM population in each province. All primary health centres providing diabetes care in each province were counted and centres were randomly selected from these. The number of primary health centres was chosen based on proportions of diabetes patients recorded in each province. Thus 15 primary health centres were the setting for the survey.

Every workday, a list of expected participants was obtained from the healthcare centres. The value of K participants depended on the number of people attending the centre each day, which varies between centres. The first K participant to be recruited into the study and who met the inclusion criteria was randomly selected by the investigator and then every Kth patient was recruited into the study. If the Kth person declined, the next person was invited. The recruitment was continued until data were collected from 1000 patients. After cleaning of the files, 25 questionnaires with missing data or unreadable handwriting were eliminated; the sample size remains 975.

A face-to-face interview was carried out by trained interviewers to collect data, including sociodemographic information, such as age, sex, place of residence, marital status, family size, level of education, and occupational status. Participants' educational levels were classified into 4 categories as follows: “Illiterate” (unable to read and write and without formal education); “Primary” (had 1 to 6 years of formal education); “Secondary” (had 7 to 12 years of formal education) and “university” (had at least 13 years of formal education). The employment status was categorized as working or not currently employed. In addition, we collected information about diabetes, such as the duration of diabetes in years, family history of diabetes (defined as having a parent or sibling with diabetes), treatment type, and complications linked to diabetes.

The inclusion criteria for this study were as follows: patients diagnosed with T2DM for 1 year or more, with an available medical file; aged at least 18 years; had an HbA1c test during the last three months; physically and mentally able to provide all data required for the study; and willing to participate in the study.

Patients with type 1 diabetes, hospitalized patients and pregnant women with diabetes were excluded from this study.

Written approval for this study was obtained from the Health Ministry, Morocco, on 3 March 2016 (reference no. 6397-3/3/2016). For the questionnaire, informed written consent was obtained from all respondents after explaining the purpose of the study, the importance of their contribution and their right to refuse participation. The data are anonymized and free of personally identifiable information.

### Anthropometric measurements and biological parameters

Height and body weight were measured for all participants by trained research staff; body weight was measured to the nearest 0.1 kg using a digital scale (Seca 877, Hamburg, Germany), and height was recorded to the nearest 0.1 cm using a wall-mounted stadiometer (Seca 216, Hamburg, Germany). Measurements were taken for each participant with light clothing and without shoes, and body mass index (BMI) was calculated as weight in kilograms divided by height in metres squared and categorized as underweight (<18.5 kg/m^2^), normal (18.5–24.9 kg/m^2^), overweight (25–29.9 kg/m^2^) and obese (≥ 30 kg/m^2^) 14.

Waist circumference was also measured to the nearest 0.5 cm, and abdominal obesity (AO) was defined as waist circumference (WC) ≥102 centimetres in men and ≥88 centimetres in women^14^.

For biological indicators, the most recent HbA1c measurements (if not exceeding 3 months prior) were extracted from medical patients' records. According to the ADA, we defined glycaemic status as good glycaemic control if HbA1c <7% and poor glycaemic control if HbA1c ≥ 7%^16^,^17^.

### Statistical analysis

Statistical analysis was carried out using Statistical Package for Social Sciences (Version 19.0, SPSS, Inc) Software. Data are described as the mean ± standard deviation (SD) for continuous variables and proportions for categorical variables. Numerical variables were analyzed using the student t-test. The association between overweight/obesity and the determinant factors considered were researched through bivariate logistic regression analysis and then all significant variables in the bivariate analysis (p< 0.05) were considered in the multivariate logistic regression model to determine independent factors associated with being obese or overweight. For each statistical test used, the test is considered significant when P-value (degree of significance) is less than 0.05.

## Results

### Socio-demographic, clinical and anthropometric characteristics

The socio-demographic, clinical and anthropometric characteristics of participants are presented in table 1.

**Table 1:** Socio-demographic, clinical and anthropometric characteristics of T2DM patients

Variables	Categories	n (%)/ Mean± SD
Sex	Men Women	255 (26) 724 (74)
Age (years)	≤40 41–50 51–60 ≥61	91 (9.5) 199 (20.7) 336 (35) 335 (34.9)
Area of residence	Urban Rural Peri-urban	744 (76) 112 (11.4) 123 (12.6)
Marital status	Single Married Divorced Widow/er	53 (5.4) 657 (67.2) 60 (6.1) 209 (21.3)
Educational level	Illiterate Primary Secondary University	651 (66.5) 154 (15.7) 129 (13.2) 45 (4.6)
Occupation	Unemployed/Housewife Employed	777(79.2) 201(20.8)
Household Members	Three and below Between 4 and 7 members Above 7 members	287(29.7) 563 (58.3) 115 (11.9)
BMI (kg/m^2^)	Under weight Normal weight Overweight Obese	11 (1.4) 225 (29.4) 309 (40.4) 220 (28.8)
Abdominal obesity	Men waist circumference (cm) Women waist circumference (cm)	95.20±16.57 100.59± 11.63
Diabetes duration (years)	-	8.55± 6.95
Glycaemic control	Mean HbA1c (%) HbA1c < 7% HbA1c ≥ 7% Fasting blood level	8.77±2.53 (245) 30.6 (556) 69.4 2.08±0.96
Type of diabetic treatment	Oral Antidiabetic (OA) alone. Insulin alone. Combination of OA and insulin. Diet only.	446 (46.1) 255 (26.4) 211 (21.8) 55 (5.4)

Women were over-represented (74 %) and the majority of the respondents (76%) was living in urban areas. The mean age was 56.19 ± 11.486 years, 9.5% were less than 40 years of age, 20.7% were 41–50 years, 35 % of the respondents represented the age group 51–60 years and 34.9 % were ≥ 61 years. Almost two thirds (66.5%) of the patients were illiterate, 15.7% had primary education, 13.2% of them had completed secondary education and only 4.6% had university education level. Over half of the study participants (67.2%) were married at the time of the study.

We noted that the prevalence of overweight, including obesity (BMI ≥ 25 kg/m^2^) was at the level of 69.2 and 28.8% of the respondents were obese (BMI ≥ 30 kg/m2). The remaining proportions of participants (29.4%) were normal weight, while only 1.4% were underweight. Regarding AO, measured by WC, the results of this study showed that the mean value of WC was significantly higher in women (100.59 ± 11.63 cm) than in men (95.20 ±16.57 cm) (t= -3.287; P<0.001). Concerning the duration of diabetes, the mean duration was 8.55± 6.95 years. The average fasting plasma glucose and HbA1c of the subjects were higher than the ADA treatment goals^16^ and the glycaemic control measured by HbA1c showed that 69.4% of the patients were classified as having poor glycaemic control (HbA1c ≥ 7%). With regard to diabetic medications, 46.1% of respondents took oral medication either alone or in combination with insulin (21.8%), 26.4% were treated with insulin alone, and 5.4% were on diet only.

### Nutritional status and associated factors

Overweight and obesity were observed in 529 (69.2 %) patients. Sex, age, educational level, household members, occupation, diabetes duration, glycaemic control and type of diabetic treatment were the candidate variables for logistic regression. The following factors were statistically significant in bivariate analysis: sex, age, education level, occupation and glycaemic control (HbA1c<7%). However, by adjusting the model using multivariate logistic regression, we have found that overweight and obesity were statistically associated with female sex, age above 41 years and good glycaemic control (Table 2).

**Table 2:** Factors associated with overweight and obesity among T2DM patients

Variables	Normal weight BMI<25 n(%)	Over-weight and Obese BMI≥25 n(%)	Bivariate analysis		Multivariate analysis	
			COR(95%CI)	P-value	AOR (95%CI)	P-value
Sex Female Male	136(24.2)99(48.8)	425(75.8) 104(51.2)	2.975(2.125–4.163)	<0.001*	3.004(1.761–5.104) 1	<0.001*
Age groups (year) <40 41–50 51–60 ≥61	34(53.1) 48(31.6) 64(24.3) 85(31.5)	30(46.9) 10(68.4) 199(75.7) 185(68.5)	1.210(1.030–1.422)	0.021*	2.192(1.116–4.307) 0.992(0.600–1.638) 0.742 (0.477–1.155) 1	0.023* 0.974 0.187
Educational level Illiterate Non illiterate	139(27) 96(38.4)	375(73) 154(61.6)	1.682(1.220–2.318)	0.001*	1.005(0.656–1.541) 1	0.981
-Household members Three and below Between 4 and 7 Above 7 members	70(31.1) 134(30) 26(31.7)	155(68.9) 312(70) 56(68.3)	1.005(0.780–1.297)	0.967	-	-
Occupation Unemployed/housewife Employed	165(27.2) 70(44.3)	441(72.8) 88(55.7)	2.126(1.481–3.051)	<0.001*	1.195(0.667–2.141) 1	0.550
Diabetes duration (years) < 3 ≥3	46(31.7) 186(30.6)	99(68.3) 421(69.4)	1.052(0.712–1.553)	0.800	-	-
Glycaemic control HbA1c < 7% HbA1c≥7%	48(23.5) 144(32.8)	156(76.5) 295(67.2)	1.586(1.085–2.320)	0.017*	1.594(1.056–2.406) 1	0.027*
Type of treatment OA alone. Insulin alone OA and insulin Diet	5(29.4) 76(37.1) 107(29.2) 5(29.4)	12(70.6) 129(62.9) 256(70.5) 12(70.6)	1.243(1.015–1.524)	0.036*	2.102(0.589–7.503) 1.618(0.958–2.731) 1.398(0.857–2.279) 1	0.252 0.072 0.180

* Statistically significant at P value <0.05

COR= Crude Odds Ratio; AOR= Adjusted Odds Ratio; CI= Confidence Interval

The overweight and obesity among females were three times higher than among males (AOR= 3,004, 95% CI: 1.761–5.104, P<0.001). Regarding age, the relative probability of being overweight and obese among participants aged 41 years and above was higher than those with age below 41 years (AOR=2.192, 95% CI: 1.116–4.307, P<0.023). Concerning the glycaemic control, the relative probability of being overweight and obese among patients with good glycaemic control was higher than patients with poor glycaemic control ones (AOR=1.594, 95% CI: 1.056–2.407, P=0.027).

The prevalence of AO was higher (73.7%) among patients and was statistically significant in bivariate analysis with sex (P<0.001), age (P=0.032), occupation (P<0.001) and type of treatment P=0.016) (Table 3).

**Table 3:** Factors associated with abdominal obesity among T2DM patients

Variables	Waist circumference		Bivariate analysis		Multivariate analysis	
	Normal n(%)	Obese n (%)	COR(95%CI)	P-value	AOR (95%CI)	P-value
Sex Female Male	38(13.6) 56(70.9)	241(86.4) 23(29.1)	2.945(1.925–4.506)	<0.001*	2.654(1.507–4.671) 1	0.001*
Age groups (year) <40 41–50 51– 60 ≥61	4(26.7) 21(28) 28(23.1) 38(28.4)	11(73.3) 54(72) 93(76.9) 96(71.6)	1.205(1.016–1.429)	0.032*	2.565(1.187–5.544) 1.320 (0.830–2.100) 0.962 (0.655–1.414) 1	0.117 0.241 0.844
Educational level Illiterate Non illiterate	58(21.6) 36(40.4)	211(78.4) 53(59.6)	0.809(0.576–1.137)	0.222	-	-
Household members Three and below Between 4 and 7 Above 7 members	25(25) 57(26.6) 12(29.3)	75(75) 175(73.4) 29(70.7)	1.734(0.566–0.950)	0.199	-	-
Occupation Unemployed/ housewife Employed	57(19.1) 37(61.7)	241(80.9) 23(8.7)	2.518(1.590–3.988)	<0.001*	0.721 (0.389–1.336) 1	0.298
Diabetes duration (years) < 3 ≥3	19(31.1) 75(25.6)	42(68.9) 218(74.4)	1.285(0.871–1896)	0.206	-	-
Glycaemic control HbA1c < 7% HbA1c ≥ 7%	29(11) 49(24)	71(89) 155(76)	1.205(0.838–1.734)	0.315	-	-
Type of treatment OA alone Insulin alone OA and insulin Diet	52(29.7) 24(28.9) 16(17.2) 9(52.9)	123(70.3) 52(71.1) 77 (82.8) 8(47.1)	1.246(1.042–1.489)	0.016*	2.794(0.958–8.154) 2.927(1.031–8.757) 1.820(0.612–5.411) 1	0.060 0.048* 0.282

* Statistically significant at P value <0.05

COR= Crude Odds Ratio; AOR= Adjusted Odds Ratio; CI= Confidence Interval

By adjusting the model using multivariate logistic regression, a statistically significant difference was found in AO to female sex (AOR=2.654, 95% CI: 1.507–4.671, P<0.001) and insulin treatment (AOR=2.927, 95% CI: 1.031–8.757, P=0.048).

## Discussion

The main goal of weight management is to ensure optimal glycaemic control, avoiding the diabetes complications. In line of this, the present study has assessed the prevalence of overweight and obesity and its associated factors among T2DM patients. The results showed that 69.2 % of respondents were overweight (BMI ≥ 25 kg/m^2^) of which 28.8% of them were obese (BMI ≥ 30 kg/m^2^). These findings are in accordance with that found in Yemen in 2014, reporting that 58.8% of patients with T2DM were overweight and 28.8% of them were obese^18^. Similarly, our data are equivalent to those reported by Tseng CH. (2007) in Taiwan, another country with nutrition transition, as 65 % of the patients were overweight or obese and less than one-third had a normal BMI^19^. Equivalent data were also observed in Oman and Qatar with 60.1% and 59.7% of the diabetic patients presenting obesity, respectively^20^,^21^. However, the data of all these studies are still less high than those found in U.S. adults with diagnosed diabetes where the prevalence of overweight or obesity was 85.2%, and the prevalence of obesity alone was 54.8%^22^. Nevertheless, the prevalence of overweight and obesity showed high scores in Jordan as 91% of respondents participating in a similar study were overweight of whom 58% were obese^23^. Increased modernization and a westernized diet and lifestyle are probably behind this increased prevalence of obesity in Jordan as well as in many developing countries^24^. Furthermore, our study has shown that the prevalence of obesity in T2DM is slightly higher than in the general population taken at whole in Morocco where overweight was found to occur among 53.0% of people with the prevalence of obesity was in the range of 20.0%^7^.

In this work, we investigated also the prevalence of AO which has been found to be higher than the prevalence of obesity defined by BMI (73.7% vs. 28.8%). Unfortunately, the difference in standards adopted to characterize AO did not allow comparison with other studies. The results obtained from the multivariate logistic regression analysis indicated that female sex was significantly associated with both overweight/obesity and AO. These findings are in agreement with previous studies in Belgium, in the United Kingdom,^25^ in Saudi Arabia,^26^ in Oman,^27^ and in Yemen 18 showing that significantly higher obesity rate was noted in females in comparison to males. Several studies conducted in the Middle East and Africa showed that the factors causing this high prevalence of obesity in females rather than in males can be attributed to less physical activity, rapid urbanization, less employment and also for cultural reasons. Indeed, women who are overweight will be socially accepted as well looked and provide her with more acceptance in the community^28^.

In this study, in addition to the female sex, the multivariate logistic regression analysis showed also that overweight and obesity were significantly associated with increased age and good glycaemic control. Regarding age, this result is similar to that found in Finland in 2009 among the general population, where both in men and in women, the prevalence of obesity increased with age^29^.

For glycaemic control, our results are in line with previous studies reporting that higher BMI is associated with good glycaemic control and patient with lower BMI are poorly controlled and have low C-peptide levels, reflecting inadequate β-cell reserves^30^. In contrast, other research studies reported that overweight or obesity was associated with a significantly higher probability of having HbA1c ≥7%,^31^ a finding that may be explained by the fact that obese diabetic patients often reported irregular meal patterns, leading to poorer glycaemic control and reduced insulin sensitivity^32^. On the other hand, other research studies reported the absence of a link between BMI and glycaemic control and critics argued that the change in HbA1c was independent of the change in weight, suggesting that there was no link between the two variables^33^. This criticism arose because some individuals with higher BMI were metabolically healthy. These studies suggested that BMI should be a component of a comprehensive evaluation of the overall health status to determine the association of BMI with glycaemic control.

Regarding AO, in addition to the female sex, the multivariate logistic regression analysis showed that insulin treatment was risk factor for AO. Indeed, it is known that AO is associated with insulin resistance^34^, and that insulin treatment, causes an excess of serum insulin that can cause a constant sensation of hunger leading by consequent to a vicious circle in which overeating generates excess body fat that accumulates in the viscera leading to AO^35^. One other possible explanation is that the majority of patients with T2DM are overweight or obese at the time of diagnosis, and treatment with insulin is known to have weight gain as an adverse effect. Given these facts, therapeutic agents that target weight loss could represent another approach to the control of T2DM.

This study was the first study conducted in the BM KH region to determine factors associated with overweight and obesity among T2DM; it investigates a relatively large sample. In contrast, it has some limitations. First, the cross-sectional design of the study limits conclusions regarding the causality of the identified associations, so longitudinal studies are greatly needed; second, some factors such as dietary habits, physical activity, and psychological factors were not assessed in this study. Therefore, further studies have to be done to assess the contribution of these factors in obesity status among T2DM patients; third, the comparison of our results was difficult because studies on this topic especially in T2DM patients are scarce in Morocco.

## Conclusion

Overweight, general obesity and abdominal obesity were high among participants. The general obesity was associated with female sex and good glycaemic control, whereas abdominal obesity was associated with female sex and insulin treatment. Given the high prevalence of obesity among women, there may be additional public health benefits of targeting this population group, because their behaviors may influence the behaviors of other proximal population groups, such as their children and families. The health consequences of diabetes are compounded by overweight and obesity. However, the prevalence of overweight and obesity among people with diabetes has not been monitored regularly given this fact weight management should receive a higher priority in the management of diabetes.

## References

[1] Massi-Benedetti M, CODE-2 Advisory Board (2002). The cost of diabetes Type II in Europe: the CODE-2 Study. Diabetologia.

[2] International Diabetes Federation (IDF) (2015). diabetes atlas.

[3] International Diabetes Federation (2003). Diabetes atlas.

[4] Amuna P, Zotor FB (2008). Epidemiological and nutrition transition in developing countries: impact on human health and development. Proc Nutr Soc.

[5] Benjelloun S (2002). Nutrition transition in Morocco. Public Health Nutr.

[6] Tazi MA, Abir-Khalil S, Chaouki N, Cherqaoui S, Lahmouz F, Sraïri JE (2003). Prevalence of the main cardiovascular risk factors in Morocco : Results of a National Survey, 2000. Journal of Hypertension.

[7] Moroccan Ministry of Health (2018). National survey on risk factors of non-communicable diseases (STEPS).

[8] American Diabetes Association (2013). Standards of Medical Care in Diabetes-2013. Diabetes Care.

[9] Daousi C, Casson IF, Gill GV, MacFarlane IA, Wilding JPH, Pinkney JH (2006). Prevalence of obesity in type 2 diabetes in secondary care: association with cardiovascular risk factors. Postgrad Med J.

[10] Kaufman FR (2006). Diabesity: a doctor and her patients on the front lines of the obesity-diabetes epidemic.

[11] Anderson JW, Kendall CWC, Jenkins DJA (2003). Importance of weight management in type 2 diabetes: review with meta-analysis of clinical studies. J Am Coll Nutr.

[12] American Diabetes Association (2003). Standards of medical care for patients with diabetes mellitus. Diabetes Care.

[13] Williamson DF, Thompson TJ, Thun M, Flanders D, Pamuk E, Byers T (2000). Intentional weight loss and mortality among overweight individuals with diabetes. Diabetes Care.

[14] World Health Organization (2000). Obesity: preventing and managing the global epidemic; report of a WHO consultation.

[15] Ramdani N, Vanderpas J, Boutayeb A, Meziane A, Hassani B, Zoheir J (2012). Diabetes and obesity in the eastern Morocco. Mediterranean Journal of Nutrition and Metabolism.

[16] American Diabetes Association (2017). Standards of medical care in diabetes-2017. Diabetes Care.

[17] American Diabetes Association (2018). Glycemic targets: standards of medical care in diabetes-2018. Diabetes Care.

[18] Al-Sharafi BA, Gunaid AA (2014). Prevalence of obesity in patients with type 2 diabetes mellitus in Yemen. Int J Endocrinol Metab.

[19] Tseng C-H (2007). Body mass index and blood pressure in adult type 2 diabetic patients in Taiwan. Circ J.

[20] Al-Moosa S, Allin S, Jemiai N, Al-Lawati J, Mossialos E (2006). Diabetes and urbanization in the Omani population: an analysis of national survey data. Popul Health Metr.

[21] Bener A, Zirie M, Janahi IM, Al-Hamaq AO, Musallam M, Wareham NJ (2009). Prevalence of diagnosed and undiagnosed diabetes mellitus and its risk factors in a population-based study of Qatar. Diabetes Res Clin Pract.

[22] Centers for Disease Control and Prevention (CDC) (2004). Prevalence of overweight and obesity among adults with diagnosed diabetes-United States, 1988–1994 and 1999–2002. MMWR Morb Mortal Weekly Rep.

[23] Khattab M, Khader YS, Al-Khawaldeh A, Ajlouni K (2010). Factors associated with poor glycemic control among patients with type 2 diabetes. J Diabetes Complicat.

[24] Khader Y, Batieha A, Ajlouni H, El-Khateeb M, Ajlouni K (2008). Obesity in Jordan: prevalence, associated factors, comorbidities, and change in prevalence over ten years. Metab Syndr Relat Disord.

[25] Ng M, Fleming T, Robinson M, Thomson B1, Graetz N1, Margono C1 (2014). Global, regional, and national prevalence of overweight and obesity in children and adults during 1980–2013: a systematic analysis for the Global Burden of Disease Study 2013. Lancet.

[26] Alqurashi KA, Aljabri KS, Bokhari SA (2011). Prevalence of diabetes mellitus in a Saudi community. Ann Saudi Med.

[27] Al-Lawati JA, Al Riyami AM, Mohammed AJ, Jousilahti P (2002). Increasing prevalence of diabetes mellitus in Oman. Diabet Med.

[28] Al-Kandari YY (2006). Prevalence of obesity in Kuwait and its relation to sociocultural variables. Obes Rev.

[29] Lahti-Koski M, Seppänen-Nuijten E, Männistö S, Härkänen T, Rissanen H, Knekt P (2010). Twenty-year changes in the prevalence of obesity among Finnish adults. Obes Rev.

[30] Chan WB, Tong PCY, Chow CC, So WY, Ng MCY, Ma RCW (2004). The associations of body mass index, C-peptide and metabolic status in Chinese type 2 diabetic patients. Diabet Med.

[31] Bae JP, Lage MJ, Mo D, Nelson DR, Hoogwerf BJ (2016). Obesity and glycaemic control in patients with diabetes mellitus: analysis of physician electronic health records in the US from 2009–2011. J Diabetes Complicat.

[32] Ercan A, Kiziltan G (2013). Obesity-related abnormal eating behaviors in type 2 diabetic patients. Pak J Med Sci.

[33] Anari R, Amani R, Veissi M (2016). Obesity and poor glycaemic control in patients with type 2 diabetes. Int J Res Med Sci.

[34] Ramachandran A, Snehalatha C, Dharmaraj D, Viswanathan M (1992). Prevalence of glucose intolerance in Asian Indians Urban-rural difference and significance of upper body adiposity. Diabetes Care.

[35] Rodin J (1985). Insulin levels, hunger, and food intake: an example of feedback loops in body weight regulation. Health Psychol.

